# The effects of allelochemicals from root exudates of *Flaveria bidentis* on two *Bacillus* species

**DOI:** 10.3389/fpls.2022.1001208

**Published:** 2022-12-01

**Authors:** Chaofang Sun, Qiao Li, Lingling Han, Xue Chen, Fengjuan Zhang

**Affiliations:** ^1^ College of Life Science, Hebei University, Baoding, Hebei, China; ^2^ State Key Laboratory for Biology of Plant Diseases and Insect Pests, Institute of Plant Protection Chinese Academy of Agricultural Sciences, Beijing, China; ^3^ School of Life Sciences, Fudan University, Yangpu, Shanghai, China

**Keywords:** *flaveria bidentis*, root exudates, *Bacillus*, nitrogen-fixation, phosphorous-solubilization

## Abstract

To determine the allelopathic effects of root exudates from *Flaveria bidentis* on function of *Bacillus*, pot experiment was used to collect root exudates from living plants and test its allelopathic effects on function of *Bacillus frigoritolerans* and *Bacillus megaterium*, which were two dominant bacteria in the rhizosphere soil of *F. bidentis*. To obtain the allelopathic substances, the root exudates were successively extracted by N-hexane, dichloromethane, ethyl acetate, and N-butanol, and their allelopathic effects were tested. The results showed that *B. frigoritolerans* and *B. megaterium* considerably increased the concentration of available phosphorus and nitrogen, respectively, when the soil was treated with different concentrations of root exudates. Among the four organic solvent extracts, dichloromethane extracts significantly increased the abundances of *B. frigoritolerans* and *B. megaterium* and promoted their nitrogen-fixing and phosphate-solubilizing abilities. Phenol was detected in dichloromethane extracts by gas chromatograph-mass spectrometer (GC-MS). Meanwhile, phenol promoted the ability to fix nitrogen of *B. megaterium* and its growth by increasing the soil available nitrogen concentration, but phenol promoted the ability to solubilize phosphate of *B. frigoritolerans* only in 0.1mg/mL concentration. Therefore, phenol was an allelochemicals in the root exudates of *F. bidentis* that affects the growth and activities of *B. megaterium.*

## Introduction

Allelopathy directly influences the growth of surrounding plants (Schalchli et al., 2012; He et al., 2019). Phenolics and their derivatives, terpenoids, and alkaloids are the main categories of plant allelochemicals (Qu and Wang, 2008; Li et al., 2010; Lupini et al., 2018; Wang et al., 2018), which are released through different means, such as in root exudate, volatiles from leaves, and through decomposition products (Li et al., 2016). Allelochemicals can affect the soil microbial community, alter the soil nutrient cycle, and indirectly influence plant growth and interspecific competition among plant species (Zhang et al., 2021). Previous studies have discussed allelochemical identification and their means of release of various plants such as rice, *Spartina alterniflora* and *Rehmannia glutinosa* (Kong et al., 2008; Zhang et al., 2019; Yuan et al., 2020); however, few have focused on describing the mechanism through which allelochemicals affect the soil microbial community.

An increasing number of studies have shown that allelopathy plays an important role in the process of exotic plant invasion (Li et al., 2015; Adomako et al., 2019). According to the “novel weapons hypothesis”, some invasive plants can invade a new range because they possess novel biochemical weapons that function as unusually powerful allelopathic agents or as mediators of new plant-soil microbial interactions (Callaway and Ridenour, 2004; He et al., 2009). For example, root exudates of invasive plants can produce large quantities of allelopathic or antimicrobial chemicals in the soil and alter the soil microbial community (Kong et al., 2008), contributing to a successful invasion (Inderjit et al., 2011; Li et al., 2017). The interactions between invasive plants and their soil microbial community are partly based on biochemistry. For instance, *Centaurea maculosa* directly inhibits the growth of native plants by releasing secondary metabolites, such as catechins, which can significantly improve the competitiveness of invasive plants (Callaway et al., 2009). The flavonoids in the root exudates of *Sapium sebiferum* can promote the colonization of arbuscular mycorrhizal fungi (AMF), increase its biomass, and facilitate successful invasion of the invasive (Tian et al., 2021). Yet we still know little about the allelopathic agents that affect soil microbial community, further studies are required to determine the associations between novel biochemistry, soil microbial communities, and invasion success.

*Flaveria bidentis* (L.) Kuntze, has invaded more than 100 counties of 5 provinces in northern China (Xing, 2014). Extensive evidence indicates that the allelopathy of *F. bidentis* helps it to grow better than native plants (Zhang et al., 2012; Xing, 2014; Song et al., 2017), leading to loss of biodiversity and causing considerable economic loss. Zhang et al. (2012) found *F. bidentis* residues to adversely affect the early growth of cotton and impact soil fertility by releasing water-soluble allelochemicals. Root secretion is its main allelochemical release pathway (Fen et al., 2009), which mainly includes flavonoids, thiophenes, phenolics, esters, and steroids (Li et al., 2014). The invasion of *F. bidentis* disrupts the soil microbial community to its benefit (Song et al., 2017). *Bacillus* is an important group of plant growth-promoting rhizobacteria in the rhizosphere of *F. bidentis* (Khalid et al., 2004; Batoul et al., 2022). *Bacillus* can remarkably activate soil enzymes associated with carbon and nitrogen metabolism and accelerate soil circulation (Palacios et al., 2014). Its ability to fix nitrogen and solubilize phosphorus is influenced by environmental factors. For example, *Bacillus* can efficiently utilize root exudates to accelerate its growth and reproduction rate and enhance its phosphate-solubilizing ability (Valetti et al., 2018). Our previous study showed that *Bacillus megaterium* and *Bacillus frigoritolerans*, the dominant bacteria in the rhizosphere of *F. bidentis*, promote the plant’s competitive growth by increasing the phosphorus and nitrogen content of the soil (Chen et al., 2021). However, the relationship between root exudates and the ability of *Bacillus* strains to fix nitrogen and solubilize phosphorus poorly understood.

To address this knowledge gap, we characterized the effect of *F. bidentis* root exudates on soil available nutrients and on the growth and phosphate-solubilizing and nitrogen-fixation ability of *Bacillus megaterium* and *Bacillus frigoritolerans*. We asked the following questions: (1) Do the root exudates of *F. bidentis* change soil available nutrients by influencing growth and phosphate-solubilizing and nitrogen-fixation ability of *B. megaterium* and *B. frigoritolerans*? (2) If so, then which are the dominant allelopathic chemicals of the root exudates? (3) What is their action mechanism on soil nutrients?

## Materials and methods

### Soil and seeds

The soil was collected from a depth of 20-40 cm on Hebei University campus (38°52′25″N, 115°31′E), sieved (< 2 mm), and its basic properties were measured: pH (w: v=1:5) = 8.721, total nitrogen = 1.604 g/kg, organic carbon content = 10.21 g/kg, available phosphorus content = 13.3 mg/kg, nitrate nitrogen content = 14 mg/kg, and ammonia nitrogen content = 57 mg/kg. The soil was mixed with vermiculite (v/v = 1:1), purchased from Hemiao Plant and Flower Co., Ltd., Bao, China.

*F. bidentis* seeds were obtained from the Plant Protection Institute, Chinese Academy of Agricultural Sciences, Langfang, China. To eliminate the effect of pathogenic bacteria on seedling growth, the seeds were surface-sterilized in 1.5% sodium hypochlorite (NaClO) for 2 min, rinsed 5 times with sterile distilled water, submerged in 70% ethanol for 1 min, and again washed 5 times.

### Bacillus frigoritolerans and Bacillus megaterium

Bacillus frigoritolerans F60 (Accession: MN918279) and Bacillus megaterium F71 (Accession: LC430058) were selected to explore the effect of root exudates of F. bidentis on their growth and their ability to fix nitrogen and solubilize phosphate. They are the dominant Bacilli in the rhizosphere soil of F. bidentis (Chen et al., 2021).

### Experiment I: Effect of root exudates of *F. bidentis* on the density and function of *Bacillus* strains

To test whether the root exudates of F. bidentis changed soil available nutrients by influencing growth and phosphate-solubilizing and nitrogen-fixation ability of B. megaterium and B. frigoritolerans, the root exudates of F. bidentis on soil available nutrients was tested firstly, then the densities of B. megaterium and B. frigoritolerans and their phosphate-solubilizing and nitrogen-fixation ability were measured.

### Collection of root exudates of *F. bidentis*

The root exudates were collected under a natural soil environment. To initiate the experiment, the mixed soil was sterilized to eliminate the effect of soil microbial activity. Three hundred gram of the soil was put into each plastic pot (12 × 14 × 14 cm as length × width × height), a total of 220 plastic pots were used in this experiment. The surface-sterilized seeds were soaked in distilled water for 12 h and 30 seeds were planted in each pot. After germination, each pot was weeded to contain only 4 plants. The pots were randomly placed in a greenhouse for 90 days under a 10-h L: 14-h D photoperiod at 25 °C.

**Figure 1:**
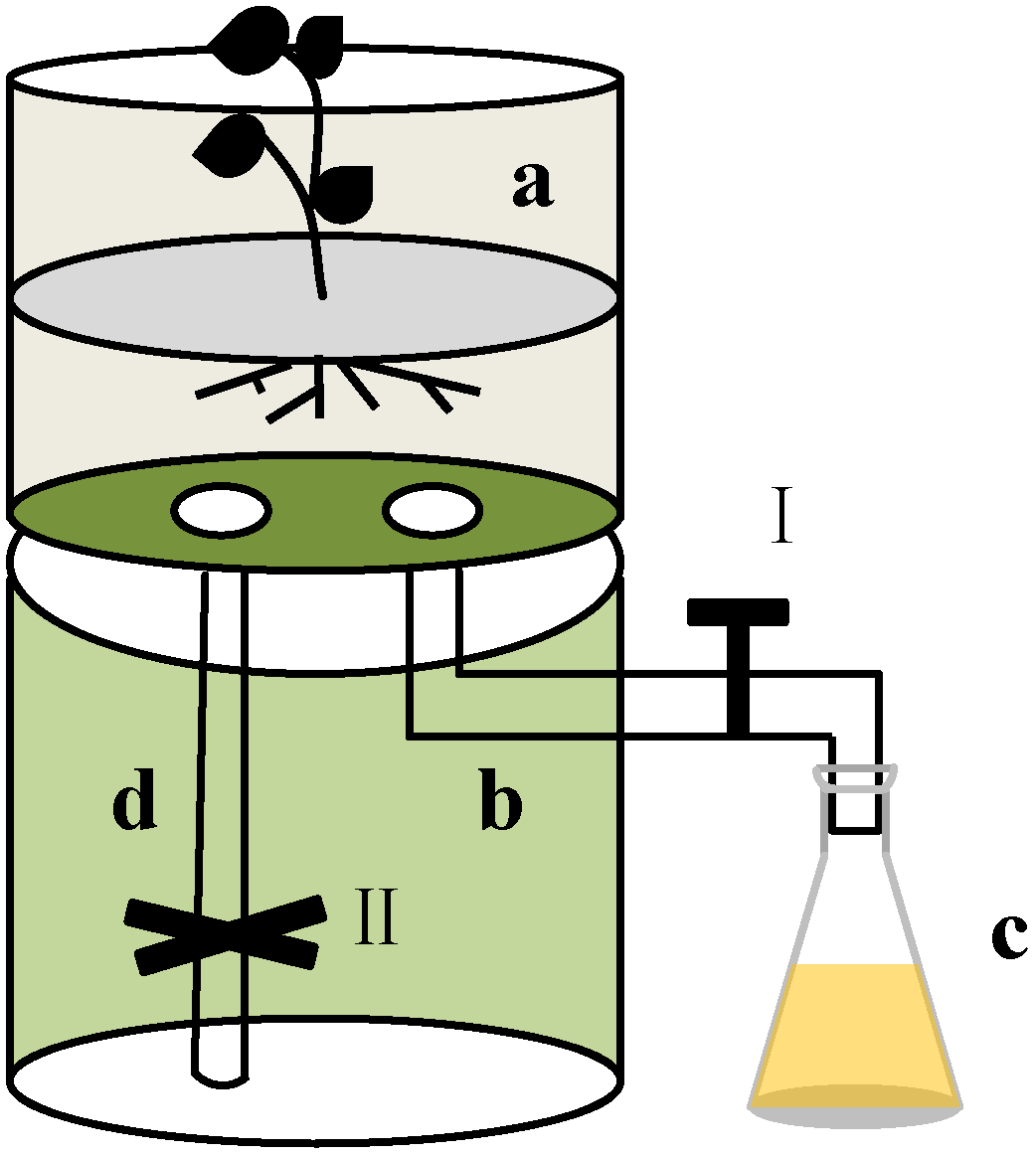
The device for collecting root exudates from *F. bidentis.* The device was made of PVC tube (160mm diameter and 40cm height) and was divided into two parts. The plant grew at the top of the device **(A)**. There is a hose **(B)**, which is connected to a triangular flask **(C)**, in the lower part. I was the control valve. During the growth, sterile water was poured in the top of the device **(A)** and the root exudates were collected in a triangular flask **(C)**. We can also collect the remaining root exudates through hose **(D)**, and the process was controlled by valve II.

To test the effect of root exudates of F. bidentis on soil available nutrients, a Polyvinyl chloride (PVC) tube was used to collect the root exudates from F. bidentis (**Figure 1**). The 4 plants from each pot were carefully transferred into the top of the Polyvinyl chloride (PVC) tube (a). Five hundred mL sterile water was poured in each PVC tube (a) slowly, and the root exudates were exported from the bottom hose of this device (b) to the conical flask(c). About 120 L root exudates were collected from each PVC tube. Then the root exudates were concentrated to 1/60 of its volume using a rotary evaporator at 30 °C. The concentrated solution was filtered through a 0.22 μm microporous membrane to remove any microorganisms. The filtered fluid was divided into three parts and stored at 4 °C.

### Preparation of bacterial suspension

The *B. megaterium* and *B. frigoritolerans* strains were taken from glycerin tubes and incubated on nutrient agar plates (1% peptone, 0.3% beef extract, 0.5% NaCl, and 1.5% agar) separately at 30°C for 24 h to obtain individual colonies. The colonies of activated *Bacilli* were selected with an aseptic toothpick and incubated in 1 mL of nutrient liquid medium culture in a 1.5 mL centrifuge tube. After shaking at 180 rpm for 24 h at 37°C, the liquid was transferred into a triangular flask containing 100 mL of nutrient liquid broth medium and shaken at 180 rpm for 24 h at 37°C. The optical density of the suspension was adjusted to approximately 1.0 (O.D. at 600 nm) by diluting it with sterile distilled water. The population count of *Bacillus* was maintained at 10^8^ CFU/mL.

### Effect of *F. bidentis* root exudates on strain density, the ability of *B. megaterium* and *B. frigoritolerans* to fix nitrogen and solubilize phosphate

The densities of *B. megaterium* and *B. frigoritolerans* were determined to explore the effects of different treatments on their growth for each fresh soil sample using suspension dilution techniques on agar plates with the nutrient broth medium (Du et al., 2020). Soil samples (1 g) were collected, and 9 mL of distilled water was added. After being shaken to homogeneity at 200 rpm for 1 day, the suspension was heated in a hot water bath at 90°C for 10 min. After 12 h standing, the supernatant was serially diluted from 10^-2^ to 10^-5^. Up to 0.1 mL was taken from each aseptic dilution using a flattened micropipette and added to nutrient agar plates. The plates were incubated at 37°C for 12 h. The amounts of *B. megaterium* and *B. frigoritolerans* were estimated by counting the single colonies. According to the number of colonies and colony separation, 10^-3^ of the supernatants was used to determine the densities of *Bacilli*. The colony forming units/1 g dry weight of soil (CFU/g DWs) was calculated according to the volume dilution. Each treatment was conducted in triplicate.

Fermentation medium for inorganic phosphorus-solubilizing (glucose 10.0 g, (NH_4_)_3_SO_4_ 0.5 g, MgSO_4_·7H_2_O 0.5 g, NaCl 0.2 g, Ca_3_(PO_4_)_2_ 5.0 g, KCl 0.2 g, MnSO_4_ 0.03 g, FeSO_4_ 0.003 g, distilled water 1000 mL, pH: 7.0 to 8.0) and organic phosphorus-solubilizing (glucose 10.0 g, (NH_4_)_3_SO_4_ 0.2 g, MgSO_4_·7H_2_O 0.5 g, KCl 0.1 g, MgCl_2_ 6H_2_0 5.0 g, calcium phytic acid 2.0 g, distilled water 1000 mL, pH: 7.0 to 8.0) were sterilized at 121 °C for 30 min. The activated bacterial suspension (1 mL each) was put into 50 mL fermentation medium for inorganic and organic phosphorus-solubilizing and cultured at 30 °C in a shaking incubator at 180 rpm for 5 days. The fermentation broth was centrifuged at 8000 rpm for 15 min in a sterile centrifuge tube. Subsequently, the culture supernatant was harvested and used for measuring the amount of phosphate produced by isolates, using the molybdenum-antimony-D-iso-ascorbic-acid colorimetry method at 720 nm in a spectrophotometer.

Ashby’s nitrogen-free agar (Mannitol 10.0 g, KH_2_PO_4_ 0.2 g, MgSO_4_·7H_2_O 0.2 g, NaCl 0.2 g, CaSO_4_ 0.1 g, CaCO_3_ 5 g, distilled water 1000 mL, pH: 7.0 to 7.2) was sterilized at 121 °C for 30 min. Bacterial suspension (1 mL) was put into 50 mL of Ashby medium for nitrogen-fixing and cultured at 30 °C in a shaking incubator at 180 rpm for 5 days. The fermentation broth was transferred to digestion tube. After acid digestion with H_2_SO_4_-H_2_O_2_, the suspension was harvested and was measured for the amount of nitrogen produced by isolates, in accordance to the Nessler’s reagent colorimetric method at 420 nm in a spectrophotometer.

### Changes of soil available nutrients

To test the root exudates and *Bacillus* species on soil available nutrients the changes of soil available phosphorous and nitrogen were measured. In specific, the filtered fluid was divided into 3 concentrations levels: C_1_ (20×the volume of concentrated root exudates), C_2_ (40×the volume of concentrated root exudates), and C_3_ (60×the volume of concentrated root exudates). In total, 40 Petri plates of 10 cm diameter were prepared for the experiment. Sterilized soil (30 g) was put into each Petri plate. Ten Petri plates of 10 cm diameter were prepared for each concentration. One mL of root exudate of each C_1_, C_2_, and C_3_ were put into their respective plates; 1 mL of sterile water (C_0_) was put in the control Petri plate. After being homogeneous mixed with soil, the 4 concentration levels were divided across 2 types of treatment: inoculation with either *B. megaterium* or *B. frigoritolerans* suspension. Five times were repeated for each concentration of *B. megaterium* or *B. frigoritolerans* suspension in the experiment. After the soil was homogenized, the Petri dishes were sealed with Parafilm membranes and incubated at 25 °C for 14 days. Soil available phosphorous was measured using the molybdenum antimony colorimetry method (Quevauviller, 1998), and nitrogen by the automatic chemical analyzer (Smart-Chem200).

### Experiment II: Effect of different extraction phases of *F. bidentis* root exudates on soil available nutrients, and density and function of *Bacillus* Strains

To analysis of allelopathic substances, 4 organic reagents that have different polarity were used to extract the root exudates and their allelopathic effects were tested. Specially, the concentrated solution of root exudates of *F. bidentis* was diluted to 500 mL with sterile water and extracted with 200 mL of each of the 4 organic reagents with increasing polarity: N-hexane, dichloromethane, ethyl acetate, and N-butanol. The 4 components were obtained. Each organic phase was concentrated under reduced pressure, dried, and weighed. The 4 components were then mixed with methanol to obtain mother solutions (1 mg/mL for each organic phase). Each organic phase repeated 3 times. Mother solutions (1 mL each) were added to sterilized Petri dishes; 1 mL of methanol was used as the control. After methanol volatilization, 30 g of sterilized soil and 1 mL of sterile water were added into the Petri dishes. *B. megaterium* or *B. frigoritolerans* (1 mL) suspension was added into the sterilized soil; 1 mL of liquid medium was added to the control. After the soil was homogenized, the Petri dishes were sealed with Parafilm membranes and put into dark constant temperature incubator at 25 °C for 14 days. Each treatment was replicated 5 times.

After the *Bacillus* strains were treated with different extraction phases, their densities and abilities to fix nitrogen and solubilize phosphate were determined by the method described previously in Experiment I.

### Experiment III: Identification of the allelochemicals that affect the function of *B. megaterium* and *B. frigoritolerans* strains

Gas chromatography mass spectrometry (Agilent GC7890B MS7000C, HP-5MS UI column 30 m × 0.25 mm × 0.25 μm) was performed to determine the allelopathic organic components in root exudates of *F. bidentis*. The analyses were conducted in splitless injection mode; the septum purge flow was 3 mL/min. Carrier gas was helium (constant flow rate of 2.5 mL/min), while nitrogen was collision gas (constant flow rate of 1.5 mL/min). Samples (1 mL) were introduced into the GC system. The initial oven temperature was set at 50°C, then increased at 20°C/min up to 150°C and held for 1 min, then at a rate of 1 °C/min up to 250°C, and then at 1°C/min up to 300°C. The total run time was 157 min. The temperature of injector was set at 250 °C, the transfer line at 285 °C, and the temperature of ion source was 230°C. The ions were generated using electron ionization (EI) ion source at 70 eV and scanned mass range of 50 to 500 amu. Primary identification of compounds was based on the Mass spectrum library (NIST14 library, Scientific Instrument Services, Inc., Ringoes, NJ, USA). Some compounds were further identified according to the mass spectrum and retention time of the standard compounds.

The determined compound of phenol was treated with methanol solution and divided into 5 concentrations: 0.01, 0.05, 0.1, 0.2, and 0.4 mg/mL. In total, 25 Petri plates of 10 cm diameter in this treatment were prepared, sterilized soil (30 g) was put into each Petri plate. Compounds of different concentrations (1 mL) were added to Petri plates and mixed with the soil; 1 mL of sterile water was put on Petri plates as the control. The 5 concentration level compounds were performed to inoculate either *B. megaterium* or *B. frigoritolerans* suspension. After homogenization, the Petri dishes were sealed with Parafilm membranes and put into dark constant temperature incubator at 25 °C for 14 days. Each treatment was replicated 5 times. Soil available phosphorous and nitrogen was determined as described previously.

After the *Bacillus* strains were treated with different compounds, their densities and abilities to fix nitrogen and solubilize phosphate were determined as described previously.

### Statistical analyses

For non-targeted data analysis, the normality and homoscedasticity of raw data were verified. The non-normally distributed data were normalized by $\;\text{Y}=\text{log}10\sqrt{\text{x}+1}$ . All statistical analyses were performed in SPSS 21.0 (IBM). Multiple comparisons of significant differences in the ability to fix nitrogen or solubilize phosphate of *B. megaterium* and *B. frigoritolerans* and their density between different treatments were examined by one-way analysis of variance (ANOVA). The difference was considered significant at confidence level of 0.05.

## Results

### Experiment I:Effect of root exudates of *F. bidentis* on the density and function of *Bacillus* strains

**Figure 2:**
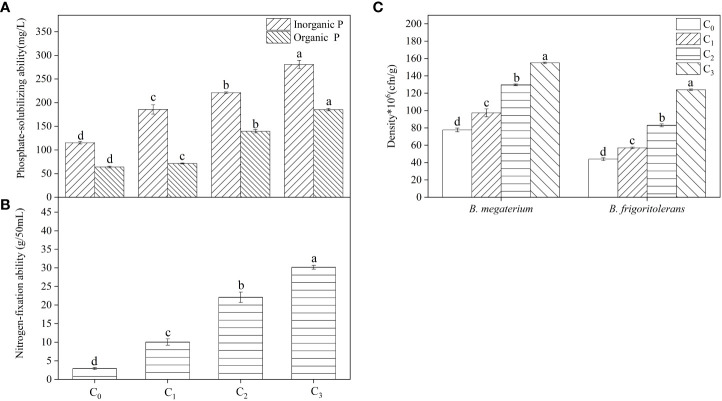
The effect of root exudates on the phosphate-solubilizing ability of *B*. *frigoritolerans* **(A)** and the nitrogen-fixation of *B*. *megaterium* **(B)** and their densities **(C)**. C_0_, sterile water; C_1_, 20×the volume of concentrated root exudates; C_2_, 40×the volume of concentrated root exudates; C_3_, 60×the volume of concentrated root exudates. Different lowercase letters above the data bar indicate that the strain with different concentrations root exudates has significant differences at the *p* < 0.05 level. The “*”indicated that density of the Bacillus strains multiplied by 10^6^.

After pot treatment with different concentrations of root exudates, changes in soil available phosphorus and nitrogen concentrations were different in each strain (**Table 1**). *B. frigoritolerans* significantly increased the concentration of soil available phosphorus (*F* = 35.814, *P* < 0.001), and *B. megaterium* increased the concentration of soil available nitrogen (*F* = 85.329, *P* < 0.001); the maximum concentrations increased by 1.45 and 2 times, respectively. The *F. bidentis* root exudates promoted their phosphorus solubilization or nitrogen fixation activity (**Figures 2A, B**) and increased the number of *Bacillus* colonies (**Figure 2C**, all *P* < 0.001). The ability of *B. frigoritolerans* to solubilize phosphorus enhanced significantly with the increasing concentration of root exudates. The ability to solubilize organic and inorganic phosphorus reached 185 and 293 mg/L in C_3_ treatment, respectively (**Figure 2A**, all *P* < 0.001). The nitrogen-fixation ability of *B. megaterium* also showed an obvious upward trend with increased concentration of root exudates. Maximum nitrogen fixation ability was 30.18 g/50 mL (**Figure 2B**, *F* = 389.4, *P* < 0.001).

**Table 1:** Effect of *Bacillus* on soil available nutrients after being treated with root exudates of *F. bidentis*.

	Strains	C_0_	C_1_	C_2_	C_3_
AP	*B. megaterium*	28.14 ± 2.95a	32.39 ± 2.93a	32.02 ± 3.61a	32.64 ± 3.47a
	*B. frigoritolerans*	25.01 ± 1.63c	28.25 ± 2.57c	42.42 ± 3.24b	61.24 ± 3.27a
AN	*B. megaterium*	114.36 ± 6.51d	165.8 ± 1.56c	253.4 ± 15.28b	342.5 ± 13.93a
	*B. frigoritolerans*	94.3 ± 1.88a	96.53 ± 0.98a	98.47 ± 0.8a	99.04 ± 0.83a

AP, Available phosphorus; AN, Available nitrogen; C_0_, sterile water; C_1_, 20×the volume of concentrated root exudates; C_2_, 40×the volume of concentrated root exudates; C_3_, 60×the volume of concentrated root exudates. Different lowercase followed after values in the same line indicate that the same strain with different concentrations root exudates has significant differences at the p <0.05 level. The values are shown as mean ± standard deviation.

### Experiment II:Effect of different extraction phase of root exudates on soil available nutrients, the density and the function of *Bacillus* strains

**Figure 3:**
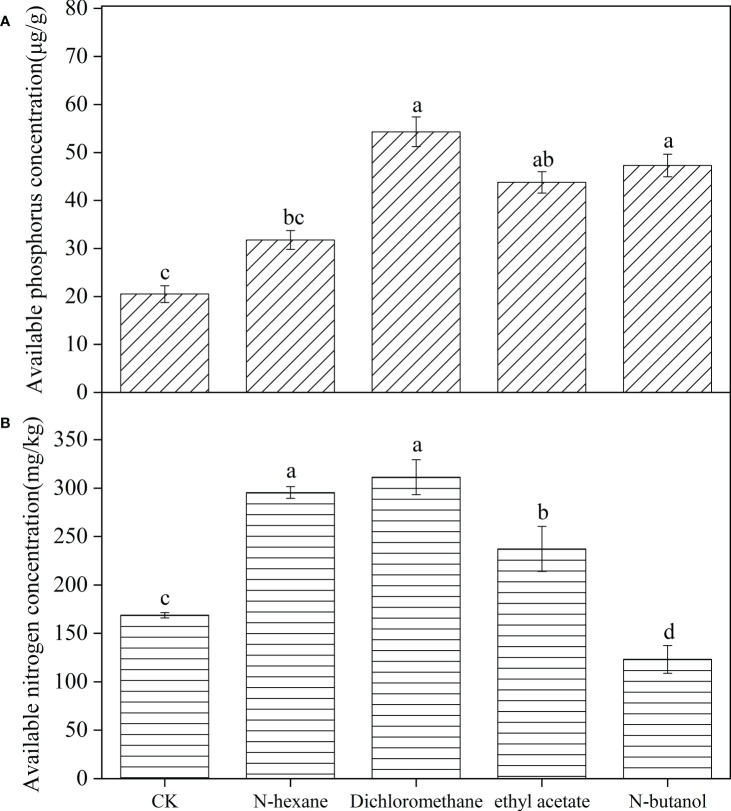
Effect of *B*. *frigoritolerans* on soil available phosphorus **(A)** and effect of *B*. *megaterium* on soil available nitrogen **(B)**, after being treated with different extraction phases of root exudates. Different lowercase letters indicate that the same strain with different extracts treatments has significant differences at the *P* < 0.05 level.

**Figure 4:**
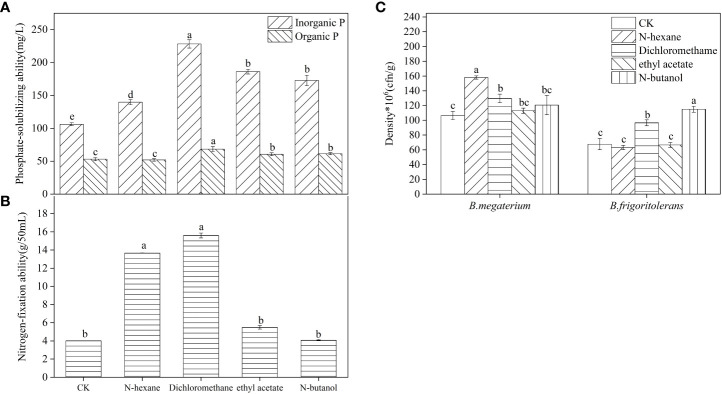
The effect of different extraction phases of root exudates on the phosphate-solubilizing ability of *B*. *frigoritolerans* **(A)** and the nitrogen-fixation of *B*. *megaterium* **(B)** and their densities **(C)**. Different lowercase letters above the data bar indicate that the strain with different extracts treatments has significant differences at the *P* < 0.05 level. The “*”indicated that density of the Bacillus strains multiplied by 10^6^.

The concentration of available phosphorus in the soil inoculated with *B. frigoritolerans* was significantly increased by 164.88%, 113.56%, and 130.68%, with respect to the control, when treated with the 3 extracts (dichloromethane extract, ethyl acetate extract, and n-butanol extract) respectively (**Figure 3A**, *F* = 539.044, *P* < 0.001). Its ability to solubilize organic and inorganic phosphorus was also significantly increased when treated with the extracts (all *P* < 0.001). The concentrations were the highest in dichloromethane extracts treatment (**Figure 4A**). The number of *B. frigoritolerans* significantly increased by 69.94% and 42.86% in N-butanol and dichloromethane extracts respectively, as compared to the control (**Figure 4C**, *F* = 47.85, *P* < 0.001).

The available nitrogen concentration in the soil that was inoculated with *B. megaterium* significantly increased by 75.1%, 84.5%, and 40.6%, relatively to the control, when the soil was treated with N-hexane, dichloromethane, and ethyl acetate extraction, respectively, while it decreased when the soil was treated with N-butanol (**Figure 3B**, *F* = 58.471, *P* < 0.001). The ability of *B. megaterium* to fix nitrogen significantly increased in N-hexane and dichloromethane extract treatments (**Figure 4B**, *F* = 2819.77, *P* < 0.001). The number of *B. megaterium* in the soil treated with N-hexane and dichloromethane extract significantly increased by 48.6% and 21.94% respectively, as compared to the control (**Figure 4C**, *F* = 16.077, *P* < 0.001).

In general, the effect of dichloromethane extracts on the functional characteristics and growth of *B. frigoritolerans* and *B. megaterium* was higher than that of any other reagent. Therefore, the extraction phase of dichloromethane was further separated and identified by GC-MS to explore the related allelochemicals.

### Experiment III: Identification of the allelochemicals that affect the function of *B. frigoritolerans* and *B. megaterium* strains

More than 38 compounds, belonging to esters, alcohols, ketones, hydrocarbons, amines, nitriles, phenolics, and acridines, were identified in the dichloromethane extract (**Supplementary Figure 1A** and **Supplementary Table S1**). The concentration of phenol (C_6_H_5_OH) was the highest among phenolic compounds (**Supplementary Figures 1B**, **C**). Moreover, phenolics are reported as the most important allelopathic substances in soil-plant-environment interaction (Quideau et al., 2011). Therefore, the compound of phenol was conducted to measure the allelopathic effects on *Bacillus* strains.

**Figure 5:**
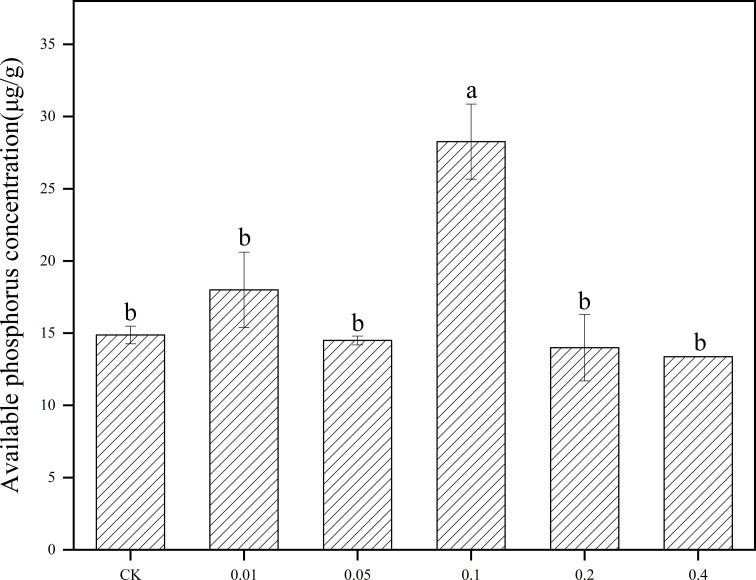
Effect of *B*. *frigoritolerans* on soil available phosphorus after being treated with different concentration phenol treatments. Different lowercase letters indicate that the same strain with different concentration of phenol treatments has significant differences at the *P* < 0.05 level.

**Figure 6:**
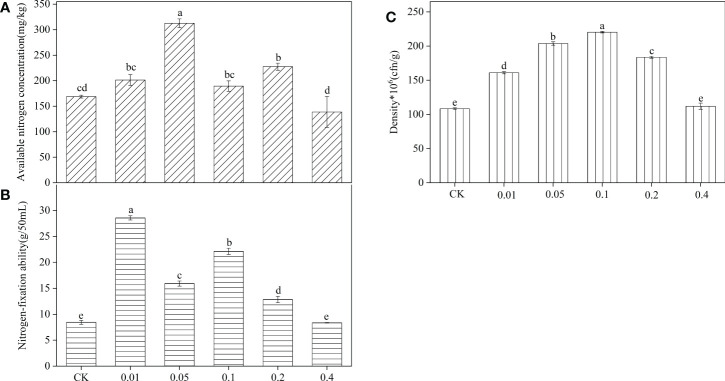
Effect of *B*. *megaterium* on soil available nitrogen after being treated with different concentration phenol treatments **(A)**, and the nitrogen-fixation ability **(B)** and density **(C)** of *B*. *megaterium*. Different lowercase letters indicate that the same strain with different concentration of phenol treatments has significant differences at the *P* < 0.05 level. The “*”indicated that density of the Bacillus strains multiplied by 10^6^.

After *B. frigoritolerans* was treated with different concentration of phenol, it was noted that the concentration of soil available phosphorus was significantly increased only in 0.1mg/mL phenol-treated soil (**Figure 5**). After *B. megaterium* was treated with different concentrations of phenol, it was observed that the available nitrogen concentration in the soil significantly increased by 85.27% and 34.83% when the soil was treated with 0.05 mg/mL and 0.2 mg/mL phenol, respectively (**Figure 6A**, all *P* < 0.001). The ability of *B. megaterium* to fix nitrogen significantly increased at 0.01 mg/mL, 0.05 mg/mL, 0.1 mg/mL and 0.2 mg/mL levels of phenol treatments (**Figure 6B**, all *P* < 0.001). The number of *B. megaterium* significantly increased in phenol treatments of 0.01 mg/mL, 0.05 mg/mL, 0.1 mg/mL, and 0.2 mg/mL, with respect to the control (**Figure 6C**, all *P* < 0.001). The experimental evidence indicated that phenol is an allelopathic substance affecting the ability of nitrogen fixation of *B. megaterium.*

## Discussion

Our results indicate that (i) root exudates of *F. bidentis* can alter the growth and function of *B. frigoritolerans* and *B. megaterium*, and (ii) phenol can promote nitrogen-fixation ability of *B. megaterium* and its density by increasing the soil available nitrogen concentration. The successful invasion of certain exotic species depends on some beneficial bacteria, such as nitrogen-fixing and phosphate-solubilizing bacteria (Sun et al., 2021). *Bacillus* is one of the rhizosphere-promoting bacteria (Qin et al., 2022). Members of this genus are known to improve plant growth, directly or indirectly, through nutrient acquisition (Abadi et al., 2020). Previous studies have shown that *Bacillus* diversity differed in exotic and native plant rhizosphere (Chen et al., 2022; Du et al., 2022). *Bacilli* are beneficial to exotic invasive species because of their capacity to enhance plant nutrient uptake, produce growth-promoting compounds, and prevent colonization of root surfaces by pathogenic fungi (Chen et al., 2021). We also find that the growth and functions of *Bacillus* strains are affected by the allelochemicals released from plant root exudates. This suggests that the invaders influencing the nutrient cycling by altering soil microbial community is a potential mechanism of successful plant invasion.

*Bacilli* are common soil-dwelling bacteria (Traxler and Kolter, 2015), and *F. bidentis* recruits specific *Bacillus* species in its rhizosphere, such as *B. frigoritolerans* and *B. megaterium*. Moreover, these species are also dominant in the rhizosphere of native species (Chen et al., 2022). However, the phosphorus-solubilizing ability of *Bacillus* strains in the rhizosphere is different in invasive and native plants during competitive growth (unpublished). The allelochemicals released from the root exudates can alter nitrogen-fixing and phosphorous-solubilizing abilities of *Bacillus* strains. The difference in root exudates between invasive and native plants may lead to the difference in available nutrients in the soil. The increase in soil available nutrients of exotic plants can benefit its invasion.

Exotic plant species can release allelochemicals to support its successful invasion. Generally, the allelopathic substances can directly affect the functions of specific microbe to alter soil nutrients. For example, the quercetin and strigolactones, two chemical signals in the root of exotic species, can stimulate the growth and root colonization of AMF to enhance its ability to absorb soil nutrients (Yu et al., 2022). But it is also possible that root exudates and allelopathic substances alter soil nutrients and then affect the functions of microbes. The wheat recruits beneficial bacteria that suppress the pathogen through solubilizing nutrients (Habib et al., 2020). In our study, we find that phenol can increase the density and nitrogen-fixation ability of *B. megaterium* by altering soil available nitrogen concentration, suggesting that phenol may be an important allelochemicals that affects soil *Bacillus* community and their functions. Studies showed that phenolic compounds are carbon-based secondary metabolites and are widely present in plants (Chacon and Arnesto, 2006; Ibrahim et al., 2011). Phenolic compounds play an important role in soil-plant-environment interactions (Quideau et al., 2011; Arafat et al., 2020). Root exudates released from exotic plant can change the concentration of phenolic compounds in the soil (Xue et al., 2021). Meanwhile, phenolic compounds can responsible for changes in soil N cycling, changing NH+ 4immobilization and gross nitrification (Castells et al., 2004). The experimental evidences in our systems suggest that the soil available nutrients and the nitrogen-fixing ability of *B. megaterium* increased with the increase in concentration of root exudates. Some studies have also demonstrated that the root exudates of *Ageratina adenophora*, maizes, and apple trees significantly increase the abundance of *Bacillus* in the soil and the soil available nitrogen (Sun et al., 2021; Wang et al., 2021; Wang et al., 2022). The interactions among plant-microbes-nutrient may give positive feedback on the exotic plants (Arafat et al., 2020).

The study suggests phenol is an important allelochemical that increases soil available nitrogen by altering the growth and nitrogen-fixing ability of *B. megaterium*. However, phenol has little impact on soil available phosphorus when the soil is inoculated with *B. frigoritolerans*. This suggests different allelopathic compounds are functionally selective for specific strains (Santonja et al., 2018; Ankati and Podile, 2019). Furthermore, studies showed that plants can recruit beneficial bacteria by releasing specific compounds in the root exudates, ultimately formatting a self-promoting mechanism (Habib et al., 2020; Sun et al., 2021). For example, *Arabidopsis thaliana* can specifically promote the activity of three bacterial species in the rhizosphere soil to resist pathogenic bacteria and promote its growth (Berendsen et al., 2018). The rhizosphere of tomato can change the activity of specific PGPR populations (*Bacillus* and *Flavisolibacter* spp.) to improve the heavy metal toxicity of plants (Zhou et al., 2022). Our study further shows the specific relationship between allelopathic compounds and the activities of rhizospheric beneficial bacteria of *F. bidentis*. To discover the mechanism of the effect of root exudates of *F. bidentis* on soil available phosphorus, the allelopathic compound from the root exudates and its effect on the growth and solubilizing phosphorus ability of *B. frigoritolerans* need to be further explored.

## Conclusion

Root exudates of *F. bidentis* increased soil available phosphorus and nitrogen levels and the abilities of *B. frigoritolerans* and *B. megaterium* specifically to solubilize phosphorus and fix nitrogen. Phenol, which was detected in root exudates of *F. bidentis*, could promote the nitrogen-fixing ability of *B. megaterium* and its density by increasing the soil available nitrogen concentration. Therefore, phenol was an allelochemicals in the root exudates of *F. bidentis* that affects the growth and activities of and *B. megaterium.*

## Data availability statement

The original contributions presented in the study are included in the article/**Supplementary Material**. Further inquiries can be directed to the corresponding authors.

## Author contributions

All authors contributed to the designed, writing, and revision of this manuscript and made intellectual contributions. All authors contributed to the article and approved the submitted version.

## Funding

We thank the fund from the National Natural Science Foundation of China (Grant No. 31972343 and Grant No. 32272562), Hebei National Natural Science Foundation (C2022201032), National Key Research and Development Program of China (2022YFC2601100).

## Conflict of interest

The authors declare that the research was conducted in the absence of any commercial or financial relationships that could be construed as a potential conflict of interest.

## Publisher’s note

All claims expressed in this article are solely those of the authors and do not necessarily represent those of their affiliated organizations, or those of the publisher, the editors and the reviewers. Any product that may be evaluated in this article, or claim that may be made by its manufacturer, is not guaranteed or endorsed by the publisher.
