# Supplementary material for: The effects of allelochemicals from root exudates of Flaveria bidentis on two Bacillus species
Source: Front Plant Sci. 2022 Dec 1;13:1001208. doi: 10.3389/fpls.2022.1001208 (PMC9751909; doi:10.3389/fpls.2022.1001208)
Supplement: Supplementary file 1 [file DataSheet_1.docx]

Supplementary Material
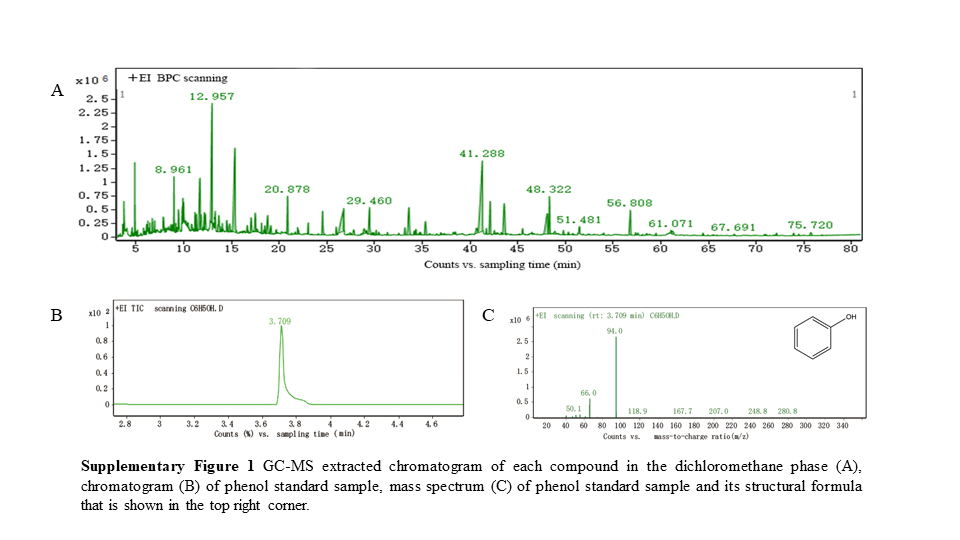


**Supplementary Figure 1** GC-MS extracted chromatogram of each compound in the dichloromethane phase (A), chromatogram (B) of

phenol standard sample, mass spectrum of phenol standard sample and its structural formula (C).

Supplementary Table S1 GC-MS identification for main substances in dichloromethane extract phase of *F. bidentis* root exudates

| R. Time | Compounds Name | Relative content (%) | Molecular Formula | Similarity (%) |
| --- | --- | --- | --- | --- |
| 41.288 | Oleic acid amide | 10.22 | C_18_H_35_NO | 87.13 |
| 15.349 | 3,3,4,6-tetramethyl-1-indanone | 8.79 | C_13_H_16_O | 80.69 |
| 12.957 | 5-oxane tricyclic [4.4.0.01, 4] dec-2-ene-7-ol, 4, 6, 10, 10-tetramethyl -, (1R, 4R, 6S, 7R) - rel - (9 chlorine) | 8.11 | C_13_H_20_O_2_ | 71.28 |
| 11.687 | 1,4-bis(1-hydroxycyclopentyl)-1,3-butadiyne | 4.41 | C_14_H_18_O_2_ | 74.79 |
| 26.733 | 2 (1H) - pyrazinone, 5-chloro-6 - (2, 4-dichlorobenzyl) -1- (2-19 alkane) -3- (1 H-pyrazole-1-yl) | 3.86 | C_15_H_26_O_4_ | 68.44 |
| 43.576 | Benz(a) acridine | 3.22 | C_17_H_11_N | 73.54 |
| 48.111 | Dimethyl (bis ([5-methyl-2 - (1-methyl vinyl) hex-4-ene-1-yl] oxyl) silane) | 3.01 | C_22_H_40_O_2_Si | 67.24 |
| 33.609 | 3-fluoro-4-[1-hydroxy-2-(propan-2-ylamino)ethyl]benzene-1,2-diol | 2.74 | C_11_H_16_FNO_3_ | 74.03 |
| 48.322 | Dioctyl phthalate | 2.23 | C_24_H_38_O_4_ | 77.09 |
| 42.116 | Stearamide | 2.21 | C_18_H_37_NO | 69.81 |
| 29.46 | Tolunitrile | 2.1 | C_18_H_33_N | 82.6 |
| 20.878 | Benzene-1, 4-diol, 2, 3-dimethyl-5-trifluoromethyl | 1.97 | C_9_H_9_F_3_O_2_ | 81.04 |
| 56.808 | Cis-13-Docosenoamide | 1.93 | C_22_H_43_NO | 78.24 |
| 4.863 | 2，3，4-Trimethyl dextran | 1.55 | C_9_H_16_O_5_ | 63.34 |
| 18.787 | α - isooctyl alcohol | 1.55 | C_15_H_24_O | 75.47 |
| 8.961 | 2,5-Diisopropylphenol | 1.43 | C_12_H_18_O | 67.47 |
| 9.904 | 2，5-Octadecadiynoic acid methyl ester | 1.39 | C_19_H_30_O_2_ | 78.13 |
| 24.552 | 2，4，7，14-Tetramethyl-4-vinyl-tricyclic[5.4.3.0（1，8）]tetradecane-6-alcohol | 1.38 | C_20_H_34_O | 76.83 |
| 13.665 | 5，7-hexadecanodienoic acid methyl ester | 1.34 | C_17_H_26_O_2_ | 78.51 |
| 17.484 | Aristolochic acid-1（10）-ene-9-ol | 1.25 | C_15_H_24_O | 76.41 |
| 35.327 | 1-Benzylisoquinoline | 1.12 | C_16_H_13_N | 72.69 |
| 17.055 | Card-20(22)-enolide,3,5,14,19-tetrahydroxy- | 1.07 | C_23_H_34_O_6_ | 76.6 |
| 3.719 | Phenol | 1 | C_6_H_5_OH | 63.64 |
| 12.242 | 3-hydroxy - a - violene | 0.92 | C_13_H_20_O_2_ | 74.57 |
| 9.443 | 3-(2,6,6-trimethyl-cyclohexen-1-yi) acrylaldehyde | 0.86 | C_12_H_18_O | 77.94 |
| 7.843 | 1, 7, 7-trimethyl-bicyclic [2.2.1] heptane-2, 3-ester | 0.83 | C_10_H_18_O_2_ | 73.65 |
| 11.803 | 2-methoxy-6, 10-dimethyl-dodecyl-2E, 6Z, 10Z-trienoic acid, 12-acetoxy -, methyl ester | 0.83 | C_18_H_28_O_5_ | 74.3 |
| 12.148 | 1H-1, 2, 4-triazolium, 5-[(1, 3-benzodioxacyclopentene-5-methyl) thiol]-3- (4-methoxy phenyl) | 0.83 | C_14_H_22_O_2_ | 76.07 |
| 11.313 | 2-Methylene-5α-cholestan-3β-ol | 0.66 | C_28_H_48_O | 76.61 |
| 14.514 | (4S,4aR,6R,8aR)-4a-hydroxy-4,8a-dimethyl-6-prop-1-en-2-yi-3,4,5,6,7,8-hexahydro-2H-naphthalen-1-one | 0.66 | C_15_H_24_O_2_ | 76.76 |
| 40.675 | 15,15 '-bi-1, 4, 7, 10, 13-pentaoxacyclohexadecane | 0.6 | C_22_H_42_O_10_ | 79.06 |
| 9.835 | 2,5-Octadecadiynoic acid methyl ester | 0.59 | C_19_H_30_O_2_ | 80.94 |
| 19.11 | 1-Heptatriacotanol | 0.58 | C_37_H_76_O | 79.55 |
| 26.766 | 2 (1H) - pyrazinones, 5-chloro-6 - (2, 4-dichlorobenzyl) -1- (2-19 alkane) -3- (1 H-pyrazole-1-yl) | 0.58 | C_15_H_26_O_4_ | 69.49 |
| 51.481 | (2S, 2's) -2, 2 '- bis [1, 4, 7, 10, 13-pentaoxacyclopentane | 0.55 | C_20_H_38_O_10_ | 77.75 |
| 14.808 | (6, 8-dihydroxymethyl-4-isopropyl - 7-methylene - bicyclic [3.2.1] oct-1-yl) -methanol | 0.52 | C_15_H_26_O_3_ | 77 |
| 27.815 | 1-Heptatriacotanol | 0.5 | C_37_H_76_O | 80.17 |
